# A Systematic Analysis of Expression and Function of RAS GTPase-Activating Proteins (RASGAPs) in Urological Cancers: A Mini-Review

**DOI:** 10.3390/cancers17091485

**Published:** 2025-04-28

**Authors:** Hao Song, Guojing Wang, Guoqiang Gao, Huayu Xia, Lianying Jiao, Kaijie Wu

**Affiliations:** 1 Department of Urology, The First Affiliated Hospital of Xi’an Jiaotong University, Xi’an 710061, China; song_hao@stu.xjtu.edu.cn (H.S.); 11306699@stu.xjtu.edu.cn (G.W.); gaoguoqiang@stu.xjtu.edu.cn (G.G.); huayv.xia@gmail.com (H.X.); 2 Department of Biochemistry and Molecular Biology, School of Basic Medical Sciences, Xi’an Jiaotong University Health Science Center, Xi’an 710061, China; jiaoly@xjtu.edu.cn

**Keywords:** RASGAPs, RAS signaling pathway, cancer therapy, urological cancers

## Abstract

The RAS signaling pathway is one of the most commonly dysregulated pathways in urological cancers. This pathway can be regulated by RASGAPs, which catalyze the hydrolysis of RAS-GTP to RAS-GDP. As such, the loss of RASGAPs can promote the activation of the RAS signaling pathway. Dysregulation of RASGAPs significantly contributes to the progression of urological cancers, including prostate cancer, bladder cancer, and renal cell carcinoma. Furthermore, alterations in RASGAP expression may influence sensitivity to chemotherapy, radiotherapy, and targeted therapies, suggesting their potential as therapeutic targets. Despite the challenges involved, a deeper understanding of the complexity of the RAS signaling network, along with the evolution of personalized medicine, holds promise for delivering more precise and effective treatment options targeting RASGAPs in urological cancers.

## Simple Summary

The RAS signaling pathway, a key communication system in tumors, is often disrupted in urological cancers, such as prostate, bladder, and kidney cancers. Normally, RASGAPs act as “off switches” for this pathway by breaking down a molecule (RAS-GTP) that keeps the pathway active. When RASGAPs are lost or dysfunctional, the RAS pathway becomes overactive, fueling cancer growth and spread. Research shows that dysregulation of RASGAPs may also affect the function of treatments for urological cancers, such as chemotherapy, radiation, or targeted drugs. This suggests that restoring or targeting RASGAPs might improve outcomes for patients. While the mechanism behind this improvement is complex, advances in personalized precision medicine could unlock new therapies that exploit RASGAPs to fight these cancers more effectively. In short, understanding RASGAPs’ function in the RAS signaling pathway offers hope for smarter, more precise treatments in the future.

## 1. Introduction

**Figure 1:**
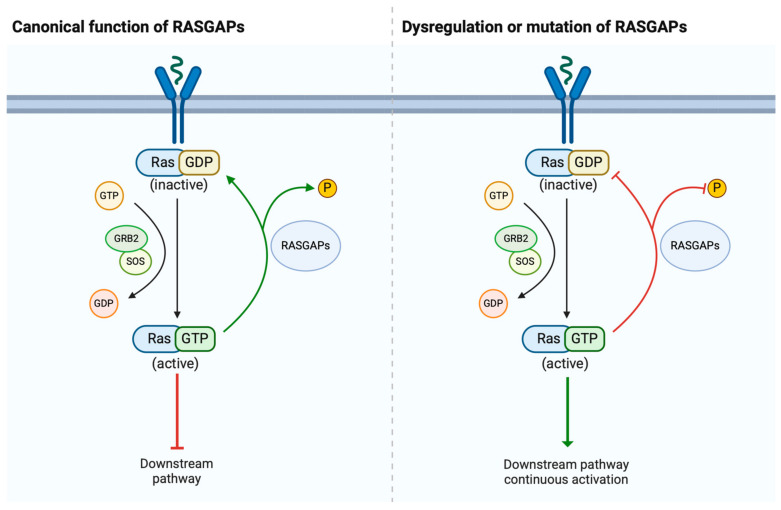
Summary of the function of RASGAPs in the RAS signaling pathway. In the pathway, RASGAPs are a class of important proteins that play a crucial negative regulatory role in the RAS signaling pathway. RASGAPs accelerate the hydrolysis of GTP on RAS proteins, converting them from an active state (GTP-bound state) to an inactive state (GDP-bound state), thereby shutting off the transmission of RAS signals.

Urological cancers, including prostate cancer (PCa), bladder cancer (BCa), and renal cell carcinoma (RCC), represent significant global health challenges due to their prevalence and impact on patients’ quality of life [1]. These malignancies often share common molecular features, such as aberrant signaling through the RAS pathway, a critical regulator of cell growth, differentiation, and survival [2,3]. Upon activation, the RAS pathway triggers downstream signaling cascades, such as the MAPK and PI3K-Akt pathways, which are involved in driving tumor initiation and progression. The RAS gene family includes three main members (KRAS, NRAS, and HRAS), and they encode small GTPase proteins that act as molecular switches, cycling between an active GTP-bound state and an inactive GDP-bound state [4,5]. The balance of RAS activity is tightly controlled by two classes of regulatory proteins, namely guanine nucleotide exchange factors (GEFs) and RAS GTPase-activating proteins (RASGAPs). GEFs, such as SOS (son of sevenless) typically promote RAS activation, but RASGAPs, such as Disabled-2 Interacting Protein (DAB2IP), enhance the intrinsic GTPase activity of RAS, leading to its inactivation [6,7] (Figure 1).

Emerging research has shown that RASGAPs play crucial roles in controlling cell differentiation, proliferation, and survival [8]. This review focuses on the expression and function of RASGAPs in urological cancers, highlighting their functional significance, their dysregulation in tumor progression, and their potential as therapeutic targets.

## 2. Structure and Physiological Roles of RASGAPs

### 2.1. Structure of RASGAPs

The RAS signaling pathway is central to many oncogenic processes. Early experiments have demonstrated that RAS GTPase activity could be accelerated by specific cytosolic proteins, leading to the discovery of RASGAPs [9]. These proteins are shown to bind RAS and enhance its intrinsic GTPase activity, converting RAS-GTP to the inactive RAS-GDP form, thereby acting as critical negative regulators of RAS activity [10]. Aberrations in RASGAPs usually lead to dysregulated RAS signaling, causing excessive cell proliferation and tumorigenesis [11]. Thus, RASGAPs provide critical insights into the regulation of RAS signaling, highlighting their importance in cellular homeostasis.

Since the identification of the first RASGAP in the late 1980s, 14 types of RASGAPs have been discovered, with each exhibiting tissue-specific expression and distinct regulatory mechanisms [12]. The 14 main members include Neurofibromin 1 (NF1), DAB2-interacting protein (DAB2IP), SYNaptic GTPase Activating Protein 1 (SYNGAP1), Ras activator-like protein 1/2/3 (RASAL1/2/3), GTPase-activating protein 1/2/3/4/5 (RASA1/2/3/4/5), and IQ motif containing GTPase-activating protein homologue 1/2/3 (IQGAP1/2/3). All 14 of these RASGAPs share a conserved GAP functional domain, which binds to RAS proteins and directly modulates the RAS activity. Additional domains mediate protein–protein or protein–lipid interactions [13,14]. In the case of H-RAS inactivation, the GAP domain engages a key catalytic residue E61 in catalysis by extending an arginine side chain to the Ras active site [15,16]. However, mutations in critical residues, such as E61 or G12/13, prevent GTP hydrolysis, locking RAS in its active state to perpetuate oncogenic signaling [17,18].

Furthermore, research has also revealed that different RASGAP proteins exhibit variations in their distribution and function. Some RASGAPs regulate not only the RAS pathway but also participate in the modulation of other RAS-independent pathways [7].

### 2.2. Physiological Roles of RASGAPs

RASGAPs play crucial roles in cellular signaling, particularly in the regulation of RAS proteins. The tumor suppressor function of certain RASGAPs is primarily linked to their GAP domain’s GTPase enzymatic activity and their ability to negatively regulate RAS activity, which is central to control cell proliferation, differentiation, and survival [5].

The tumor suppressor function of RASGAPs reveals their importance in cellular homeostasis and highlights them as potential targets for therapeutic intervention in cancers where RAS signaling is aberrantly activated. However, it is important to note that not all RASGAPs necessarily act as tumor suppressors. Some studies suggest that their specific roles may depend on the context and particular cellular environment. For example, certain cancers may exploit RASGAP-mediated signaling for cancer cell survival or invasion [19,20]. In the IQGAP family, the expression of IQGAP2 is reduced and acts as a tumor suppressor in most solid cancer types, while IQGAP3 is overexpressed and functions as an oncogene [21]. The dual roles of RASGAPs complicate their therapeutic targeting, highlighting the importance of understanding the specific cancer context in which RASGAPs are dysregulated.

Moreover, it has been observed that certain RASGAP proteins possess broader regulatory roles beyond the RAS pathway. They may influence multiple signaling networks, contributing to diverse biological outcomes. For example, some RASGAPs have been shown to engage in pathways related to cell adhesion, cytoskeletal dynamics, and even vesicle trafficking, independent of their canonical function in deactivating RAS [22,23,24]. The multiple functions of RASGAPs reveal the complexity of cellular regulation and underscore their importance in the full spectrum of activities associated with individual RASGAP proteins.

## 3. Dysregulation of RASGAPs Promotes Urological Cancers

**Figure 2:**
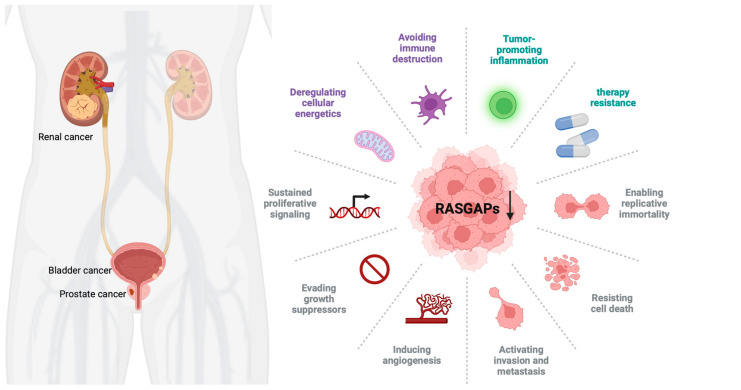
The oncogenic effects of RASGAP downregulation in urological cancer. RASGAP downregulation activates the RAS signaling pathway by inhibiting RAS from an active state (GTP-bound state) to an inactive state (GDP-bound state), thereby promoting urological cancers’ malignancy. The interplay between RASGAP dysregulation and oncogenic processes highlights their therapeutic relevance in urological malignancies.

The RAS pathway is highly conserved and regulates a variety of cellular processes. Dysregulation of this pathway, especially through mutations in RAS genes, can lead to uncontrolled cellular proliferation and cancer development [25]. In urological cancers, specific mutations in HRAS, KRAS, and NRAS have been linked to tumor formation and aggressive cancer phenotypes [26,27]. While mutations in RAS genes are less frequent in urological cancers, dysregulation of RASGAPs plays a critical role in the development and progression of these cancers. Members of the RASGAP family exhibit tumor-suppressive roles in urological tumors, providing new insights into their treatment. However, among the RASGAP family members, only some of them have been closely associated with the occurrence, development, and prognosis of urological tumors, with certain specific mechanisms remaining unclear. Therefore, future research must continue to explore the relationships between other RASGAP family members and urological tumors to offer more options for precise treatment. The three major urological cancers (PCa, BCa, and RCC) can exhibit dysregulation of the RAS pathway. However, the specific RASGAPs involved (e.g., DAB2IP in PCa, NF1 in BCa, RASAL2 in RCC) and their functional roles may vary. Although these cancers share common RASGAP pathway components, such as DAB2IP, the extent of pathway involvement and its functional significance can differ due to variations in molecular context, tumor biology, and genetic alterations (Figure 2).

### 3.1. RASGAPs and Prostate Cancer

Prostate cancer (PCa) is one of the most frequently diagnosed urological cancers and one of the most frequent causes of cancer deaths in males [1]. Patients with PCa have benefitted from androgen deprivation therapies (ADTs) and small molecule inhibitors targeting the androgen receptor (AR). However, 30% of patients exhibit primary resistance to both forms of treatment, and the majority of patients progress from androgen-dependent prostate cancer (ADPC) to castration-resistant prostate cancer (CRPC) primarily due to the emergence of AR splice variant-7 (AR-V7) [28,29]. The AR remains a key driver of CRPC through aberrant activation in a low-androgen microenvironment. Understanding the molecular mechanisms of PCa has led to advancements in prognostic, diagnostic, and therapeutic approaches.

RAS signaling plays a significant role in PCa progression. While RAS mutations are rare in PCa, dysregulation of the RAS signaling pathway via alterations in upstream or downstream components, especially RASGAPs, is frequently observed. In PCa, several RASGAPs, such as RASAL3, have been found to be dysregulated. This dysregulation of RASGAPs ultimately leads to the activation of Ras signaling in carcinoma-associated fibroblasts [30].

Dysregulation of RAS signaling through alterations in RASGAPs significantly contributes to PCa progression [31]. RASGAPs, such as DAB2IP, can be dysregulated in PCa through several mechanisms, including genetic mutations, epigenetic modifications, and post-translational changes. H3K27 hypermethylation of the DAB2IP promoter, mediated by EZH2’s histone methyltransferase activity, is a common epigenetic alteration in advanced PCa, leading to its transcriptional downregulation. Loss of DAB2IP not only negatively regulates RAS activity but also GSK3β/β-catenin and NF-κB activity [32,33,34,35]. Increased activation of NF-κB in DAB2IP-deficient PCa promotes epithelial-to-mesenchymal transition (EMT) and metastasis both in vitro and in vivo. Other studies have also shown that DAB2IP knockdown promotes EMT and metastasis through targeting PROX1/HIF1α either in LAPC-4 or RWPE-1 PCa cell lines [36,37]. This evidence forecasts that DAB2IP could inhibit EMT and metastasis in PCa. As a key regulator, the loss of DAB2IP expression results in the activation of the PI3K-AKT pathway and inactivation of the ASK1-JNK pathway, leading to accelerated PCa growth in vivo [38]. Additionally, the loss of DAB2IP function may increase AR signaling, both in vitro and in vivo, and contribute to the development of CRPC [39,40]. The tumor suppressor p53 has also been found to be associated with the RASGAP pathway in PCa. PCa with mutant p53 proteins (mut p53) responds to insulin signaling by increasing cell proliferation and invasiveness in vitro. This response is mainly due to the fact that mutant p53 enhances insulin-induced AKT1 activation by binding and inhibiting DAB2IP [41]. Furthermore, other studies have demonstrated that downregulation of DAB2IP gene expression impacts PCa’s resistance to ionizing radiation (IR) [42]. DAB2IP re-expression sensitizes PCa cells to radiation and chemotherapy, underscoring the therapeutic potential of targeting RASGAPs in this malignancy. Epothilone B (EpoB), an anticancer drug, has been found to significantly increase cellular radiosensitivity in DAB2IP-deficient PCa cells [43]. Reactivating DAB2IP or inhibiting the PI3K-AKT pathway in DAB2IP-deficient PCa models has shown promise in preclinical studies [7,44]. Thus, restoring the function or expression of DAB2IP or inhibiting downstream effectors of RAS activation may provide alternative therapeutic strategies.

Moon et al. found that hypercholesterolemia was able to promote PCa metastasis by increasing IQGAP1 both in vitro and in vivo [45]. The adhesion of cancer cells to endothelial cells requires β1-integrin, and IQGAP1 regulates the transcription and expression of β1-integrin. Mechanistically, IQGAP1 functions downstream of Cdc42 to enhance β1-integrin expression through ERK/focal adhesion kinase (FAK) signaling at the protein level and by promoting myocardin-related transcription factor (MRTF)/serum response factor (SRF)-mediated transcriptional activity. This cascade ultimately upregulates β1-integrin expression, facilitating cancer cell–endothelial adhesion [46]. Xiong et al. also found that IQGAP1 is additionally associated with chemoresistance in PCa. The exocrine factor ANGPTL4 is primarily expressed in cancer-associated fibroblasts (CAFs) of PCa. Upon binding of ANGPTL4 to IQGAP1 on the membrane of PCa cells, it activates the Raf-MEK-ERK-PGC1α axis, driving mitochondrial biogenesis and oxidative phosphorylation (OXPHOS) metabolism. This mechanism promotes PCa tumor growth and confers chemoresistance [47]. IQGAP2 has been regarded as a tumor suppressor of PCa. Its expression is elevated in low-grade PCa (from prostatic intraepithelial neoplasia to Gleason 3 tumors). However, IQGAP2 is downregulated in high-grade PCa (Gleason 4-5) [48,49], and its downregulation has been shown to be positively associated with recurrence and metastasis in PCa through the activation of AKT signaling [50]. Meanwhile, IQGAP3 has been found to be positively correlated with the infiltration of B cells, macrophages, and dendritic cells, indicating its potential role as a tumor-specific antigen. Its overexpression is associated with worse overall survival rates [51].

RASA1, another RASGAP associated with aggressive PCa, has been linked to Gleason score [52]. However, the function and mechanisms of RASA1 in PCa remain incompletely understood. Meanwhile, RASAL1 has been shown to inhibit the tumorigenicity of human primary cells in both PCa and BCa [53]. Recently, RASAL2 was found to be upregulated in PCa tumors and metastatic lymph node tissues. Overexpression of RASAL2 was associated with higher PCa tumor stage, Gleason score, and poorer prognosis. Mechanistically, RASAL2 functions as an oncogene by promoting cancer cell proliferation through activation of the PI3K/AKT/cyclin D1 pathway [54]. Additionally, Tailor et al. found that RASAL2 could also function as a tumor suppressor by inhibiting cell proliferation and invasion, as well as by inducing an S phase and G2/M phase cell cycle arrest through the downregulation of TNFα [55]. RASAL3 promotes lethal PCa progression and resistance to ADT. Mechanistically, in prostatic CAFs, RASAL3 facilitates activated Ras signaling to drive macropinocytosis-mediated glutamine synthesis, fueling tumor metabolic demands. ADT further induces epigenetic silencing of RASAL3, which increases glutamine secretion from CAFs. This adaptive metabolic reprogramming sustains PCa survival and proliferation under androgen-deprived conditions, fostering therapeutic resistance and aggressive tumor growth [56].

While studies on the function of other RASGAPs in PCa are not extensive, Kachroo et al. found that downregulation of SPRED2, a RAS-regulator, is associated with a high Gleason score in PCa. SPRED2 regulates the RAS signaling pathway by modulating RASGAP’s GTPase activity through direct binding. Overexpression of SPRED2 reduced ERK phosphorylation and inhibited PCa cell proliferation and migration [57]. Meanwhile, loss-function mutations of SPRED2 have been observed in human cancers, leading to tumor progression [58].

Oligophrenin 1 (OPHN1), an indirect RASGAP containing a RhoGAP domain that enhances GTPase activity [59], is located in the same region as the AR gene, which can be amplified by ADTs. Consequently, PCa undergoing ADTs may amplify both AR and OPHN1. Liu et al. found that the overexpression of OPHN1 contributes to cell viability and enhances migration in LNCaP, 22RV1, and PC3 cells [60]. Furthermore, due to the proximity of these two genes’ location, long non-coding RNAs (lncRNAs), such as lnc-OPHN1-5, can increase sensitivity to enzalutamide (Enz) by interfering with AR mRNA expression. This finding could help to develop novel therapies to increase Enz treatment sensitivity [61].

RASGAPs have also been found to interact with phosphoprotein associated with glycosphingolipid microdomains 1 (PAG), a negative regulator of immune signaling in T lymphocytes. In this way, RASGAPs are enriched on the cell membrane to inhibit RAS activity, ultimately suppressing the ERK1/2 pathway and cyclin D1 expression [62].

### 3.2. RASGAPs and Bladder Cancer

Bladder cancer (BCa) is a common malignancy, with most cases classified as non-muscle-invasive bladder cancer (NMIBC). However, some BCa cases are characterized by high recurrence rates and progression to muscle-invasive forms (MIBC). Despite advancements in clinical treatment methods, the prognosis for BCa remains poor, particularly in the advanced stages [63,64]. RASGAPs, such as DAB2IP and NF1, have been implicated in regulating cellular growth and survival in BCa [65,66,67,68].

In addition to its essential roles in PCa, DAB2IP is a well-characterized RASGAP that regulates multiple signaling pathways in BCa. Loss of DAB2IP expression has been linked to aggressive tumor behavior in BCa [69]. In BCa, DAB2IP acts as a tumor suppressor by inhibiting the RAS and PI3K/Akt pathways. Its downregulation, mainly due to promoter hypermethylation, leads to increased cell proliferation, migration, invasion, and resistance to apoptosis, contributing to tumor growth and metastasis. Additionally, the caveolin-1 gene plays a vital role in promoter methylation of the DAB2IP gene in the progression of urinary bladder transitional cell carcinoma (TCC) from low to high potential for malignancy [70,71]. miRNA-mediated repression is another mechanism leading to DAB2IP loss in BCa. Ou et al. found that estrogen receptor beta (ERβ) induces the expression of miR-92a by binding to the estrogen-response element (ERE) in the 5′ promoter region of its host gene C13orf25. miR-92a then represses DAB2IP expression by binding to its 3′ UTR [72]. In another study, miR-92b was shown to specifically downregulate DAB2IP, promoting EMT, migration, and invasion in BCa, though it had no effect on cell proliferation [73]. Similarly, overexpression of miR-556-3p in BCa was found to downregulate DAB2IP and increase ERK1/2 phosphorylation levels [74].

As a promising biomarker, DAB2IP deficiency can promote chemoresistance and tumor recurrence in NMIBC after bladder-preserving surgery. In one study, Wu et al. observed significant downregulation of DAB2IP expression in high-grade and recurrent NMIBC specimens. This loss of DAB2IP was inversely correlated with elevated Twist1 expression and predicted poorer recurrence-free survival in patients. Mechanistically, DAB2IP deficiency in BCa cells promotes STAT3 phosphorylation and transactivation, which drives the upregulation of Twist1 and its downstream target P-glycoprotein (P-gp). This STAT3-Twist1/P-gp axis is essential for pirarubicin chemoresistance and tumor regrowth [75]. DAB2IP is also typically downregulated in BCa with a radioresistant phenotype. He et al. found that overexpression of ataxia-telangiectasia mutated (ATM), which is negatively regulated by DAB2IP, plays a central role in BCa resistance to ionizing radiation (IR). Knockdown of ATM may activate MAPK and NF-κB signaling pathways [76]. This finding suggests that ATM may be an effective target in DAB2IP-deficient BCa with an IR-resistant phenotype.

Additionally, NF1 is another important RASGAP that negatively regulates RAS signaling in BCa. NF1 deficiency has been observed in higher-grade urinary bladder transitional cell carcinoma (TCC), making it a potential biomarker [77]. High expression of NF1 could be regulated by the knockdown of Heterogeneous Nuclear Ribonucleoprotein U (HNRNPU), which enhances chemosensitivity in BCa. In T24 cancer cells with high HNRNPU expression, the knockout of HNRNPU inhibited cell proliferation, invasion, and migration. Moreover, the loss of HNRNPU promoted apoptosis and S-phase arrest in T24 cells treated with cisplatin [78]. RASAL2 expression is significantly downregulated in BCa specimens and inversely correlates with pathological grade and clinical stage. Wu et al. demonstrated that RASAL2 suppresses BCa stemness and EMT. Mechanistically, MAPK/SOX2 signaling is critical for maintaining stem-like and mesenchymal properties in RASAL2-deficient BCa cells, as inhibition of ERK activity or SOX2 knockdown reverses these phenotypes. Furthermore, RASAL2 inhibits BCa tumorigenesis and distant metastasis in vivo [79]. RASAL2 knockdown has also been shown to promote angiogenesis in BCa. Mechanistically, RASAL2 deficiency enhances AKT phosphorylation, which drives the transcriptional upregulation of ETS1 and VEGFA. This RASAL2-AKT-ETS1/VEGFA signaling axis orchestrates proangiogenic reprogramming, thereby potentiating tumor vascularization and progression in BCa [80].

Hensel et al. found that IQGAP1, a membrane of the RASGAP family, was downregulated in BCa. Its deficiency was shown to increase TGFβ signaling in BCa, suggesting that IQGAP1 has the potential to serve as both a clinical biomarker and a cancer growth suppressor [81]. Compared to normal controls, IQGAP3 was found to be highly expressed in urine samples from BCa patients. The IQGAP3/BMP4 ratio in urinary cell-free DNA has been demonstrated to be a diagnostic marker for BCa, showing high sensitivity and specificity, with a specificity for hematuria reaching 90.3% [82,83]. CDC42 and IQGAP3 were co-upregulated in both BCa tissues and cell lines. Mechanistically, CDC42 silencing reduced IQGAP3 expression and suppressed RAS/ERK signaling while concurrently inducing apoptosis and inhibiting BCa cell proliferation. Notably, IQGAP3 overexpression abolished CDC42 silencing-mediated proliferation arrest and apoptotic induction. Collectively, these findings demonstrate that CDC42 drives RAS/ERK signaling through IQGAP3 to enhance proliferation and suppress apoptosis, thereby contributing to BCa pathogenesis [84]. In contrast, IQGAP2, which is downregulated in BCa, may function to inhibit tumor proliferation, migration, and invasion by regulating the MAPK/ERK signaling pathway and cytokines [85].

Additionally, ARHGAP family genes, which share a homology with RASGAPs in their catalytic domains, have been associated with a tumor-promoting immune microenvironment in BCa. This microenvironment is characterized by a lower Th1/Th2 cell ratio, higher dendritic cell (DC) infiltration, increased Treg cell infiltration, and a T cell exhaustion phenotype [86].

### 3.3. RASGAPs and Renal Cancer

Renal cell carcinoma (RCC) accounts for over 90% of all kidney cancers and poses significant challenges due to its resistance to chemotherapy and radiation [87,88]. Currently, surgical intervention remains the primary treatment strategy for early-stage kidney tumors, while targeted therapy and immunotherapy are the main treatments for advanced stages. RCC is characterized by complex molecular mechanisms, with RAS signaling being implicated in its progression [89,90]. Understanding the molecular drivers of RCC has led to the development of targeted therapies, such as VEGF inhibitors and mTOR inhibitors, though resistance to these treatments is still a hurdle.

Emerging evidence suggests that dysregulation of RAS signaling contributes to RCC tumorigenesis. RASGAPs, particularly through the RAS/RAF/MEK/ERK and PI3K/Akt/mTOR pathways, play a central role in RCC progression [91,92]. First, DAB2IP is reported to be a key RASGAP involved in the regulation of multiple signaling pathways in RCC. Downregulation of DAB2IP in RCC has been associated with increased tumor growth and metastasis. Mechanistically, the loss of DAB2IP leads to activation of the PI3K/Akt pathway, enhancing RCC cell survival and proliferation [93]. In one study, high DAB2IP mRNA expression correlated with smaller tumor volume and better survival outcomes compared to patients with low expression. Zhou et al. found that the proline-rich domain in the C terminal (CPR) of DAB2IP could suppress AKT phosphorylation and p27 expression [93]. DAB2IP is frequently epigenetically silenced in RCC, with its loss correlating with poor overall survival. RCC cells with DAB2IP downregulation exhibit enhanced sensitivity to growth factor stimulation and resistance to small-molecule inhibitors, such as mTOR inhibitors. Mechanistically, DAB2IP deficiency leads to simultaneous activation of the ERK/ribosomal S6 kinase 1 (RSK1) and PI3K/mTOR pathways, which synergistically induce HIF-2α expression. Elevated HIF-2α subsequently represses p21/WAF1 transcription, a critical mediator of mTOR inhibitor resistance. These findings position DAB2IP as both a prognostic biomarker and a predictive marker for therapy response in RCC [94].

Moreover, the loss of DAB2IP results in elevated PARP-1 protein levels, which are associated with IR resistance in RCC, providing a new targeting strategy to improve the efficacy of radiotherapy of RCC. Mechanistically, DAB2IP acts as a scaffold to assemble a ternary complex with PARP-1 and E3 ubiquitin ligases, facilitating PARP-1 ubiquitination and proteasomal degradation, offering a new targeting strategy to enhance radiotherapy efficacy in RCC [95]. Primary cilia are microtubule-based organelles that function as sensors for physical and biochemical cues, regulating physiological and developmental processes. Their loss has been implicated in multiple cancers, including RCC. DAB2IP has been identified as a novel interactor of kinesin family member 3A (KIF3A), a core component essential for primary cilia assembly. DAB2IP stabilizes KIF3A within the axoneme, thereby maintaining primary cilia integrity. Notably, KIF3A deficiency promotes RCC tumorigenesis, establishing the DAB2IP-KIF3A complex as a critical homeostatic regulator in normal renal epithelia. Mechanistically, KIF3A interacts with the N-terminal pleckstrin homology (PH) domain of DAB2IP, which extends KIF3A’s half-life and facilitates ciliogenesis. KIF3A loss-induced ciliary disassembly drives renal tumorigenesis, highlighting the tumor-suppressive role of primary cilia stability mediated by this complex [96].

RCC, characterized by hypervascularity, is clinically managed using targeted therapies against the VEGFA/VEGFR2 signaling axis. Zhu et al. identified the lncRNA DMDRMR as a molecular sponge for miR-378a-5p, which upregulates EZH2 and SMURF1 expression. This DMDRMR/miR-378a-5p axis promotes the dual suppression of DAB2IP through EZH2-mediated transcriptional silencing and SMURF1-dependent proteasomal degradation. Consequently, DAB2IP loss activates VEGFA/VEGFR2 signaling, driving angiogenesis and conferring sunitinib resistance in ccRCC [97]. Yun et al. demonstrated that DAB2IP enhances miR-138 expression, which suppresses stem-like phenotypes in RCC by directly targeting ATP-binding cassette subfamily A member 13 (ABCA13) and EZH2. However, miR-138 downregulation in RCC is driven by DNA methyltransferase 1 (DNMT1)-mediated epigenetic silencing via promoter hypermethylation. DAB2IP may interact with DNMT1 to facilitate promoter methylation, amplifying ABCA13/EZH2-dependent pro-tumorigenic signaling [98]. Yeh et al. demonstrated that tumor-infiltrating T lymphocytes enhance RCC invasiveness by upregulating estrogen receptor β (Erβ) expression, which suppresses DAB2IP-dependent tumor-suppressive signaling. This T cell/ERβ/DAB2IP axis drives a pro-invasive cascade, suggesting that therapeutically targeting this pathway may disrupt RCC progression [99].

Second, RASAL2, another RASGAP in RCC, has been shown to inhibit angiogenesis both in vitro and in vivo by decreasing the expression of vascular endothelial growth factor A (VEGFA) through the p-GSK3β/c-FOS pathway. This study provides new insights into preventing RCC resistance to anti-vascular therapy [100].

In addition, aldehyde dehydrogenases 9 family A1 (ALDH9A1) deficiency has been linked to increased tumor proliferation, invasion, migration, and lipid accumulation in RCC. Mechanistically, reduced ALDH9A1 levels impair its cytoplasmic sequestration of nucleophosmin 1 (NPM1), leading to suppressed transcription of IQGAP2. This transcriptional repression subsequently activates the AKT-mTOR signaling pathway, which drives tumor progression and dysregulates lipid metabolism in RCC [101].

Mutations in RASGAPs also play crucial roles in regulating the RAS signaling pathway. Vanli et al. found that a point mutation in RASGAP prevented its cleavage by caspase-3, thereby enhancing RAS signaling [102]. In non-small cell lung cancer (NSCLC), NF1 co-mutation with RASA1 has been described [103]. Similarly, in breast cancer, mutations in RASAL2 have been reported to promote tumor growth, progression, and metastasis in mouse models [104]. Furthermore, loss-of-function mutations of RASA2 have been linked to increased RAS activation, melanoma cell growth, and migration in melanomas [105]. Inactivation mutations of RASGAPs promote tumorigenesis by disrupting the negative regulation of the Ras signaling pathway, potentially holding significant pathological implications in urological malignancies. However, direct evidence of RASGAP mutations in urological tumors remains limited, with most conclusions extrapolated from general mechanisms and other tumor types. Future research should focus on comprehensive genomic sequencing and functional assays to delineate the specific mutation profiles of RASGAPs in urological cancers. This would clarify their precise mechanistic roles and therapeutic potential in urological cancers. To summarize the functions of RASGAPs discussed above, Table 1 presents their expression patterns, biological effects, and mechanisms in urological cancers.

## 4. Targeting the RASGAPs Is a Significant Strategy in Cancer Therapy

The therapeutic targeting of RAS and its regulators has long been a challenge due to the “undruggable” nature of RAS [106]. RAS proteins were historically considered difficult due to their smooth surface and the high affinity of GTP binding [107]. Despite extensive research, directly inhibiting RAS has remained challenging [108]. However, recent advances in the development of direct RAS inhibitors, as well as therapies targeting downstream effectors of RAS, offer promising avenues for cancer treatment [109].

**Figure 3:**
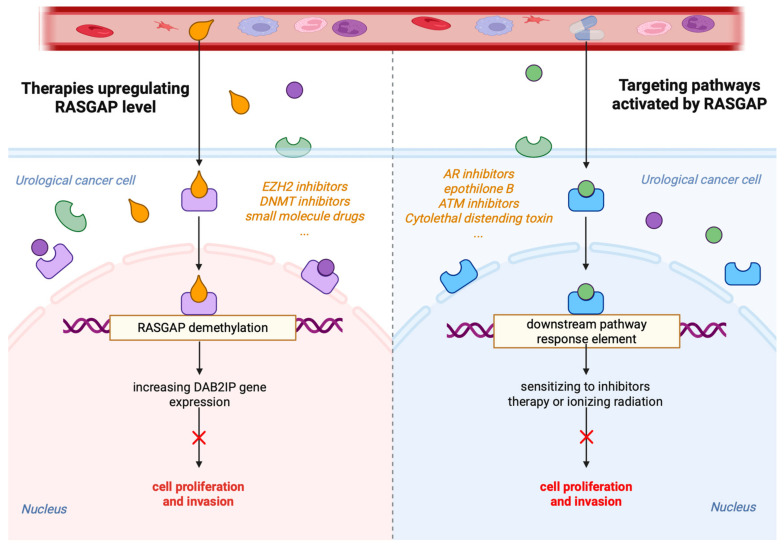
Potential therapies targeting RASGAPs in urological cancers. Therapies aimed at increasing RASGAP levels result in increased RASGAP gene expression by contributing to the demethylation of RASGAPs. Therapies targeting pathways activated by RASGAPs are shown. By targeting these pathways, the aim is to sensitize cancer cells to inhibitors, therapy, or ionizing radiation, thereby reducing cell proliferation and invasion. Consequently, these strategies seek to mitigate the effects of RAS signaling in urological cancer cells, either by enhancing the expression of RASGAPs or by directly interfering with downstream signaling pathways.

RASGAPs, which regulate multiple aspects of RAS signaling, represent attractive targets for therapeutic intervention. Restoring RASGAP function or inhibiting compensatory pathways activated by RASGAP loss may provide novel treatment strategies for urological cancers (Figure 3). One approach involves restoring the expression of downregulated RASGAPs, such as DAB2IP, using gene therapy or small-molecule drugs. Studies have shown that targeting inhibitory miRNAs can restore endogenous DAB2IP protein levels and function. However, a major limitation of RNA-based strategies in clinical applications is efficient delivery to tumor cells [110,111,112]. Significant research is currently underway to develop both lipid and non-lipid nanocarriers for improved RNA delivery [113].

Furthermore, RASGAP expression is regulated at epigenetic levels, including promoter DNA and histone hypermethylation. Since DAB2IP transcription is repressed by the epigenetic factor EZH2, targeting EZH2 with its inhibitor GSK126 could restore DAB2IP expression [114]. Similarly, inhibitors of DNA methyltransferase 3A (DNMT3A) have been proven to reduce the proliferation and survival of colorectal cancer cells by upregulating the DAB2IP protein level [115].

Alternatively, targeting pathways activated by RASGAP loss may be an effective therapeutic strategy. The negative feedback regulation between AR and PI3K/AKT signaling networks has been previously demonstrated [116]. For instance, PCa with enhanced PI3K/AKT/mTOR signaling due to DAB2IP downregulation may be more sensitive to combined treatment with PI3K inhibitors and AR pathway inhibitors, such as enzalutamide and abiraterone [117,118]. Additionally, several genotoxic drugs, including cytolethal distending toxin (CDT), epothilone B, and ATM inhibitors, have been found to resensitize DAB2IP-deficient prostate or bladder cancer cells to IR [43,119,120].

Given the complexity of RAS signaling, combination therapies targeting both RAS and its regulatory pathways may be required. Combining RASGAP modulators with inhibitors of downstream effectors, such as MEK or PI3K inhibitors, may enhance therapeutic efficacy [61]. In PCa, AR signaling supports tumor growth and survival, while PI3K/AKT activation, due to PTEN loss, drives proliferation and resistance to AR-targeted therapies. The reciprocal feedback between these pathways, where inhibition of one often leads to compensatory activation of the other, contributes to treatment resistance. These findings highlight the complexity of PCa biology and underscore the need for integrated, precision-based approaches to target interconnected pathways and improve patient outcomes [117,118].

Additionally, immunotherapies that exploit the immune-modulatory effects of RASGAPs in the tumor microenvironment are a promising avenue for future research [121]. Urological tumors with RASGAP deficiencies may possess a distinct immune landscape. Combining RAS pathway inhibitors with immune checkpoint inhibitors (e.g., anti-PD-1/PD-L1 agents) could potentially enhance antitumor immune responses and overcome therapeutic resistance.

Furthermore, the development of small molecules or peptides that mimic RASGAP function may provide a direct approach to suppressing RAS signaling. However, RAS-driven tumors are highly heterogeneous, with diverse genetic alterations influencing their response to therapy [122]. This heterogeneity complicates treatment strategies, as not all tumors will respond to the same therapeutic approach. Personalized medicine, which tailors therapies based on the specific genetic profile of each tumor, holds great promise for overcoming these challenges and optimizing therapeutic outcomes.

## 5. Perspectives on the Future of RASGAPs

While significant progress has been made in targeting the RAS signaling pathway and RASGAPs, several challenges remain. These include the development of resistance to targeted therapies [123], the heterogeneity of RAS mutations across different urological cancer types [108], and the intricate complexity of RAS signaling networks [106]. Since RASGAP-related pathways play crucial roles in normal cells, therapies targeting these pathways may induce toxicity in normal tissues. Moreover, urological tumors exhibit high heterogeneity, and RASGAP-deficient tumors may display diverse molecular characteristics and clinical behaviors. A deeper understanding of the interaction between RAS signaling and other oncogenic pathways could lead to the identification of novel therapeutic targets, particularly in PCa [124].

Another important tumor suppressor, p53, plays a vital role in preventing tumor initiation by regulating the cell cycle and DNA repair. p53 mutations, especially missense mutations, are prevalent across various cancers, including urological cancers. Mutant p53 (mutp53) can regulate key oncogenic pathways, such as PI3K/AKT/mTOR and RAS/MAPK [125]. Giulio et al. found that mutp53 enhances NF-κB activation while inhibiting ASK1/JNK via TNFα, a function that depends on its binding to and inhibition of DAB2IP in the cytoplasm [126]. As previously discussed, Elena et al. found that mutp53 promotes cell proliferation and invasiveness by interacting with DAB2IP in PCa [41]. In BCa, p53 mutations occur with high incidence [127]. However, the extensive function of DAB2IP in PCa and other urological cancers remains insufficiently understood. Further research is needed to clarify its specific mechanism.

The comprehensive review of RASGAPs in urological cancers underscores their important roles in tumor progression. While significant advances have been made in elucidating their mechanisms, several critical gaps and contradictions limit their translational application. The paradoxical roles of RASGAPs across tumor types highlight the complexity of their regulatory networks. For instance, IQGAP1 acts as an oncogene in PCa by activating Raf-MEK-ERK signaling but functions as a tumor suppressor by inhibiting TGFβ signaling in BCa. Similarly, RASAL2 inhibits MAPK/SOX2-driven stemness in BCa yet promotes PI3K/AKT activation in PCa. These discrepancies may arise from tissue-specific interactomes or post-translational modifications, but the lack of systematic comparative studies across malignancies hampers the development of unified mechanistic models.

Although DAB2IP’s tumor-suppressive functions are well documented, its application in therapy strategies of CRPC remains poorly integrated into clinical paradigms. While preclinical studies suggest DAB2IP restoration sensitizes tumors to therapy, clinical validation is still lacking. Additionally, much of the current research heavily relies on cell line studies (e.g., PC-3, T24) and xenograft models, which inadequately recapitulate human tumor heterogeneity and stromal interactions. For example, while RASAL3’s role in CAF-mediated glutamine synthesis in CRPC is mechanistically compelling, it lacks validation in patient-derived organoids or clinical cohorts. Similarly, proposed therapeutic strategies—such as using EZH2 inhibitors to reactivate DAB2IP or PI3K/AR inhibitor combinations—are supported by preclinical data but face challenges regarding delivery specificity and toxicity. The absence of clinical trials directly targeting RASGAPs represents a critical gap in the field. Moreover, while this review highlights RASGAPs’ interplay with diverse pathways (e.g., NF-κB, HIF-2α, STAT3), the hierarchical and temporal regulation of these interactions remains unclear. For instance, while DAB2IP’s suppression of PI3K-AKT and activation of ASK1-JNK in PCa is well documented, how these opposing signals are balanced in vivo remains unexplored.

To address these gaps, future efforts should focus on identifying biomarkers that predict responses to RAS-targeted therapies, understanding the resistance mechanisms, and developing more effective combination therapies [128]. Emerging technologies, such as single-cell RNA sequencing (scRNA-seq) and spatial transcriptomics, may provide deeper insights into RASGAP heterogeneity within tumor subpopulations. Additionally, prospective clinical trials correlating RASGAP expression and mutation status (e.g., DAB2IP promoter methylation) with therapeutic responses to PARP or MEK inhibitors could aid in clinical biomarker validation. Furthermore, rational combination strategies, such as pairing RASGAP modulators (e.g., EZH2 inhibitors) with immune checkpoint blockers, may enhance treatment efficacy. By integrating these approaches, we can improve our understanding of the initiation and development of urological cancers, ultimately laying the groundwork for new targeted therapies. Advances in precision medicine, coupled with deeper insights into RASGAP biology, are expected to pave the way for more personalized and effective treatments for RAS-driven cancers.

## 6. Conclusions

Urological cancers, including PCa, BCa, and RCC, demonstrate a significant reliance on aberrant RAS signaling, underscoring the importance of the RAS pathway as a therapeutic target. The RAS family of small GTPases plays an important role in regulating cellular processes, such as growth, differentiation, and survival, primarily through downstream signaling cascades, including the MAPK and PI3K-Akt pathways. Dysregulation of the RAS signaling pathway is commonly mediated by the interaction of RAS with GEFs and RASGAPs. RASGAPs are essential for inactivating RAS by accelerating the conversion of RAS-GTP to RAS-GDP, thus acting as critical regulatory checkpoints.

RASGAPs are closely associated with urological cancers, and their function in these cancers is complicated. Therefore, future research should focus on elucidating the mechanisms through which RASGAPs contribute to the development and progression of urological cancers. Such research could provide new insights into targeted therapies in urological cancers.

The distinct molecular features of RASGAPs in urological cancers provide a basis for exploring the targeting of the RAS signaling pathway therapies. Continued research into the molecular mechanisms by which RASGAPs govern RAS activation and inactivation, as well as into the specific roles of RASGAPs in various cancer contexts, will enhance our understanding of tumor biology and offer promising avenues for therapeutic intervention. However, the complexity of the RAS signaling pathway necessitates a nuanced approach, as therapeutic targeting must consider the broader signaling network and potential compensatory mechanisms within cancer cells.

## Figures and Tables

**Table 1:** The expression and function of RASGAPs in PCa, BCa, and RCC. The protein expression level of RASGAPs in urological organs (data adapted from the Human Protein Atlas database: https://www.proteinatlas.org/, accessed on 16 February 2025). The expression levels are categorized as +, ++, or +++. Note that this is a generalized representation, and specific expression levels may vary depending on the tissue and cell type.

Tumor Type	RASGAPs	Exp.	Biological Effect	Mechanism	Ref.
PCa	DAB2IP	+++	DAB2IP loss promoted PCa EMT and metastasis	Tumor suppressor: targeted GSK3β/β-catenin; targeted NF-κB signaling; targeted PROX1/HIF1α	[35,36,37]
			DAB2IP loss accelerated PCa growth in vivo	Tumor suppressor: targeted PI3K-AKT and ASK1-JNK pathway	[38]
			DAB2IP loss contributed to the development of CRPC	Tumor suppressor: targeted testosterone synthesis and AR signaling	[39,40]
			DAB2IP loss increased cell proliferation and invasiveness	Tumor suppressor: mut p53 enhanced insulin-induced AKT1 activation by binding and inhibiting DAB2IP	[41]
			DAB2IP loss promoted resistance to ionizing radiation	Tumor suppressor: enhanced DSB repair, robust G(2)-M checkpoint control, and resistance to apoptosis and	[42,43,44]
	IQGAP1	+++	IQGAP1 promoted cancer cell dissemination and metastasis	Oncogene: regulated β1-integrin via FAK and MRTF/SRF	[46]
			IQGAP1 promoted PCa tumor growth and increased chemoresistance	Oncogene: activated by ANGPTL4 in CAFs to activate Raf-MEK-ERK-PGC1α axis and drive mitochondrial biogenesis and OXPHOS metabolism	[47]
	IQGAP2	+++	IQGAP2 downregulation was associated with high Gleason score, recurrence and metastasis	Tumor suppressor: activated AKT signaling	[50]
	IQGAP3	+	IQGAP3 was positively correlated with infiltration of B cells, macrophages and dendritic cells	-	[51]
	RASA1	++	RASA1 was positively associated with aggressive PCa and Gleason score	-	[52]
	RASAL1	+	RASAL1 inhibited tumorigenicity of human primary cells	-	[53]
	RASAL2	+	RASAL2 promoted tumor cell proliferation, the transition from G1 to S phase in vitro and tumor growth in vivo	Oncogene: activated PI3K/AKT/cyclin D1 pathway	[54]
			RASAL2 overexpression inhibited cell proliferation and invasion and induced an S phase plus G2/M phase cell cycle arrest	Tumor suppressor: downregulated TNFα expression	[55]
	RASAL3	+	Epigenetic silencing of RASAL3 promoted lethal PCa growth and the development of resistance to ADT	Tumor suppressor: expressed in prostatic CAFS; activated Ras signaling and drived macropinocytosis-mediated glutamine synthesis; ADT promoted RASAL3 epigenetic silencing and glutamine secretion	[56]
BCa	DAB2IP	+++	DAB2IP-deficient BCa cells promoted chemoresistance and tumor recurrence in NMIBC	Tumor suppressor: targeted STAT3 phosphorylation and transactivation; elevated Twist1 and P-glycoprotein expression	[75]
			DAB2IP-knockdown induced BCa resistance to IR	Tumor suppressor: elevated expression of ATM; inhibited MAPK and NF-κB signaling pathways	[76]
	NF1	++	Knockout of HNRNPU enhanced cisplatin sensitivity by regulating NF1 expression	-	[78]
	RASAL1	+	RASAL1 inhibited the tumorigenicity of human primary cells	-	[53]
	RASAL2	+	RASAL2 BCa tumorigenesis and distant metastasis in vivo	Tumor suppressor: inhibited MAPK/SOX2 signaling and BCa stemness and EMT	[79]
			Knockdown of RASAL2 promoted angiogenesis in BCa	Tumor suppressor: targeted RASAL2-AKT-ETS1/VEGFA signaling axis	[80]
	IQGAP1	+++	IQGAP1 inhibited cancer growth	Tumor suppressor: regulated TGFβ signaling	[81]
	IQGAP2	+	Reduced IQGAP2 promoted tumor proliferation, migration, invasion and EMT	Tumor suppressor: regulated MAPK/ERK pathway and reduced cytokines	[85]
	IQGAP3	++	IQGAP3 inhibited apoptosis and promoted BCa cells proliferation	Oncogene: activated RAS/ERK signaling	[84]
RCC	DAB2IP	+++	DAB2IP knockdown increased cell proliferation, promoted cell cycle progression in G1/S phase	Tumor suppressor: regulated the phosphorylation level of AKT and p27	[93]
			Loss of DAB2IP enhanced RCC sensitivity to growth factor stimulation and resistance to mTOR inhibitors	Tumor suppressor: targeted ERK/RSK1 and PI3K/mTOR pathways; induced HIF-2α expression; repressed p21/WAF1 transcription	[94]
			Loss of DAB2IP elevated PARP-1 protein levels; RCC acquired IR-resistance	Tumor suppressor: DAB2IP acted as a scaffold to assemble a ternary complex with PARP-1 and E3 ubiquitin ligases, facilitating PARP-1 ubiquitination and subsequent proteasomal degradation	[95]
			DAB2IP-KIF3A complex suppressed renal tumorigenesis	Tumor suppressor: KIF3A interacted with the N-terminal PH domain of DAB2IP; extended KIF3A’s half-life and facilitates ciliogenesis	[96]
			DAB2IP loss drived angiogenesis and conferring sunitinib resistance in RCC	Tumor suppressor: DMDRMR/miR-378a-5p/DAB2IP axis; targeted VEGFA/VEGFR2 signaling	[97]
			DAB2IP-mediated miR-138 in modulating RCC stem-like phenotypes	Tumor suppressor: DAB2IP loss interact with DNMT1 to facilitate promoter methylation of miR-138; miR-138 could suppress ABCA13 and EZH2	[98]
			DAB2IP inhibition promoted RCC cell invasion	Tumor suppressor: targetd infiltrating T cells/ERβ/DAB2IP signals	[99]
	RASAL2	++	RASAL2 inhibited angiogenesis	Tumor suppressor: decreased the expression of VEGFA through p-GSK3β/c-FOS pathway	[100]
	IQGAP2	++	ALDH9A1 deficiency promoted tumor proliferation, invasion, migration, and lipid ac-cumulation in RCC through downregulating IQGAP2	Tumor suppressor; involved in ALDH9A1-NPM1-IQGAP2-AKT axis	[101]

## Abbreviations

ADPC	Androgen-dependent prostate cancer
ADTs	Androgen deprivation therapies
AKT	Protein Kinase B (also known as PKB)
AR	Androgen receptor
ARHGAP	Rho GTPase-Activating Protein
ASK1-JNK	Apoptosis Signal-regulating Kinase 1–c-Jun N-terminal Kinase
ATM	Ataxia-Telangiectasia Mutated
BCa	Bladder cancer
CAFs	Cancer-associated fibroblasts
CDT	Cytolethal distending toxin
CRPC	Castration-resistant prostate cancer
CPR	Proline-rich domain in C terminal
DAB2IP	Disabled-2 Interacting Protein
DC	Dendritic cell
DNMT1/3A	DNA Methyltransferase 1/3A
EMT	Epithelial-to-mesenchymal transition
Enz	Enzalutamide
ERE	Estrogen-response element
ERβ	Estrogen receptor beta
ERK1/2	Extracellular Signal-Regulated Kinase 1/2
GEFs	Guanine nucleotide exchange factors
GSK3β	Glycogen Synthase Kinase 3 Beta
GSK126	EZH2 inhibitor (specific compound)
HIF1α	Hypoxia-Inducible Factor 1-alpha
HIF2α	Hypoxia-Inducible Factor 2-alpha
HNRNPU	Heterogeneous Nuclear Ribonucleoprotein U
IQGAP1	IQ motif-containing GTPase-Activating Protein 1
IR	Ionizing radiation
KIF3A	Kinesin family member 3A
lncRNAs	Long non-coding RNAs
MAPK	Mitogen-Activated Protein Kinase
MEK	Mitogen-Activated Protein Kinase Kinase
MIBC	Muscle-invasive bladder cancer
mTOR	Mammalian target of rapamycin
mutp53	Mutant p53 proteins
NF-κB	Nuclear factor kappa B
NF1	Neurofibromin 1
NMIBC	Non-muscle-invasive bladder cancer
NSCLC	Non-small-cell lung cancer
OPHN1	Oligophrenin 1
PAG	Phosphoprotein Associated with Glycosphingolipid Microdomains 1
PARP-1	Poly (ADP-ribose) Polymerase 1
PCa	Prostate cancer
PD-L1	Programmed Death-Ligand 1
PI3K	Phosphoinositide 3-Kinase
PROX1	Prospero Homeobox 1
RASA1/2/3/4	Ras GTPase-Activating Protein family members
RASAL1/2/3	Ras Activator-like proteins
RASGAPs	RAS GTPase-Activating Proteins
RCC	Renal cell carcinoma
RSK1	Ribosomal S6 kinase 1
SPRED2	Sprouty-Related EVH1 Domain-Containing Protein 2
SYNGAP1	SYNaptic GTPase Activating Protein 1
TCC	Transitional cell carcinoma
TGFβ	Transforming growth factor beta
Th1/Th2	T-helper 1/T-helper 2 cells
Treg	Regulatory T cell
UTR	Untranslated region
VEGF	Vascular endothelial growth factor
